# P-752. Prevalence and risk factors for bacterial resistance in infections in hospitalized burn patients in a referral hospital in Nicaragua

**DOI:** 10.1093/ofid/ofaf695.963

**Published:** 2026-01-11

**Authors:** Marvin Castro-Averruz, Kevin Gavarrete-Rivas, Sunaya Marenco-Avilés, Guillermo D Porras-Cortés, Kevin A Sandoval-Rojas

**Affiliations:** Hospital Dr. Fernando Vélez Paiz, Managua, Managua, Nicaragua; Hospital Dr. Fernando Vélez Paiz, Managua, Managua, Nicaragua; Hospital Dr. Fernando Vélez Paiz, Managua, Managua, Nicaragua; Hospital Dr. Fernando Vélez Paiz, Managua, Managua, Nicaragua; Hospital Dr. Fernando Vélez Paiz, Managua, Managua, Nicaragua

## Abstract

**Background:**

Infection is the leading cause of death and morbidity following burn injury. The bacterial resistance has a negative impact on the clinical outcomes of these patients. The aim of this study was to determine the prevalence, the main risk factors, and clinical outcomes for patients with infected burn caused by bacteria that were resistant to different classes of antimicrobials in a hospital in Nicaragua.

**Figure 1.**
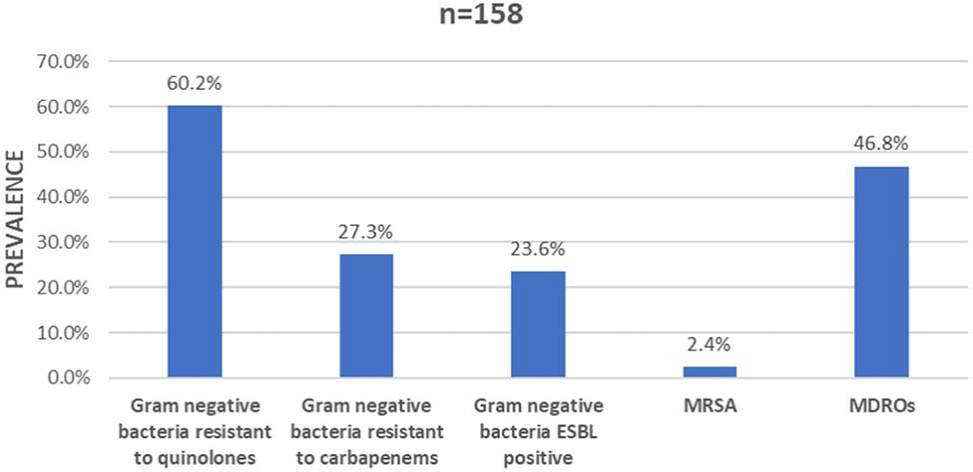
Prevalence of bacteria resistant to different classes of antibiotics in patients with infected burn

**Table 1.**
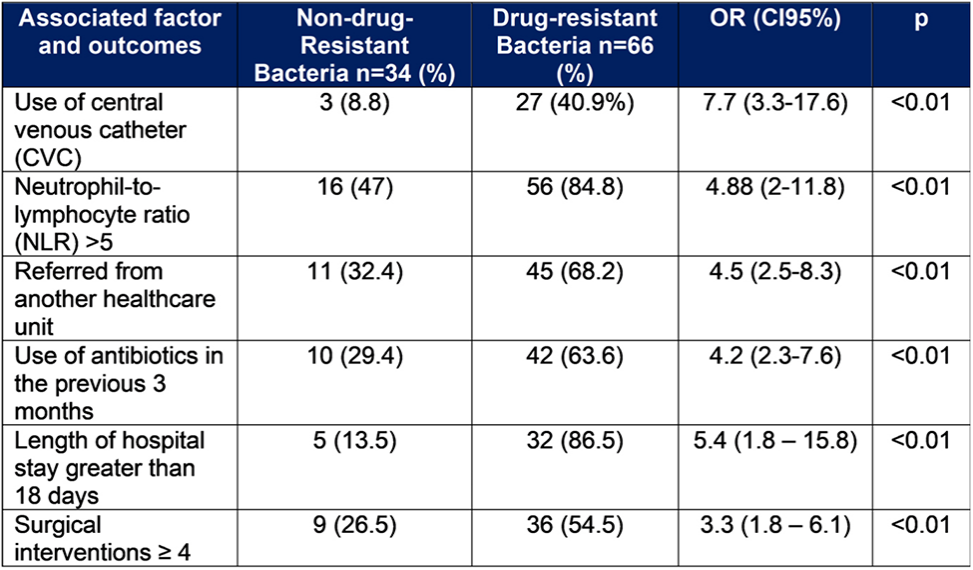
Associated factor and clinical outcome related with bacterial resistance in patients with infected burns

**Methods:**

A case-control study was conducted between January 2021 and December 2024 at Hospital Dr. Fernando Vélez Paiz in Managua (Nicaragua). Cases were patients with infected burn caused by drug-resistant organisms (DRO) and the controls were patients with infected burn caused by non-drug-resistant organisms (NDRO). Samples of tissue and exudates obtained by biopsy were processed by conventional methods as blood agar and McConkey agar, and the final identification method was Vytek 2 System. Different variables of both groups were compared.

**Table 2.**
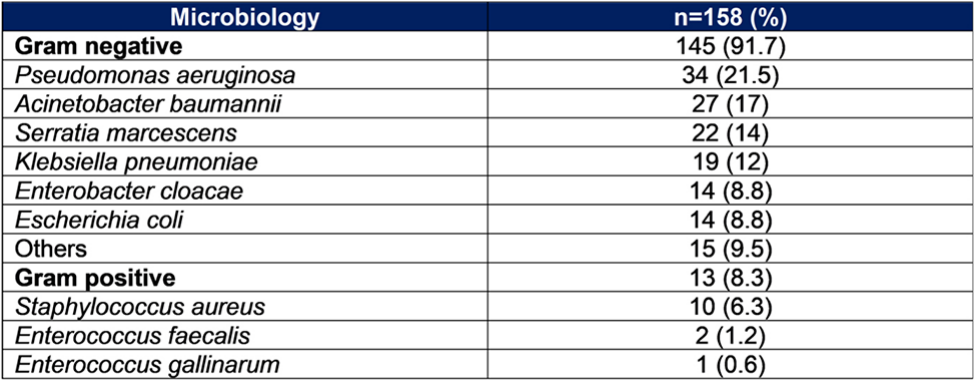
Microbiology of patients with infected burn Microbiology of patients with infected burn

**Figure 2.**
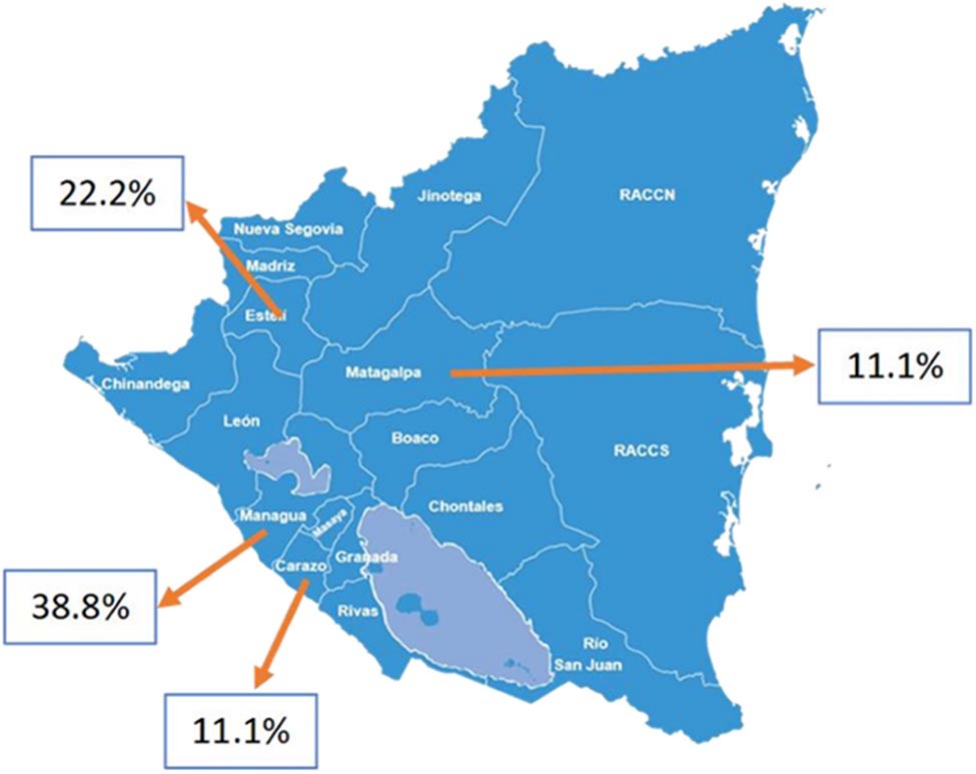
Map of Nicaragua showing the wide distribution of the prevalence distribution of drug-resistant organisms infections in burn patients referred from another healthcare unit to Hospital Dr. Fernando Vélez Paiz Hospital in the capital city of Managua.

**Results:**

A total of 158 bacteria were identified in 100 patients. Sixty-six patients had an infection with at least one resistant bacteria for a prevalence of 66%. The Gram-negative bacteria was the most prevalent bacteria group (91.7%) (Table 2). 60.2% of them were resistant to quinolones, 23.6% were producer of Extended-spectrum beta-lactamase (ESBL) and 27.7% were resistant to carbapenems (Figure 1). A total of 74 isolates were multi-drug-resistant organisms (MDRO). The main risk factors to have an infection with resistant bacteria were the use of central venous catheter (CVC) (OR:7.7; CI95%: 3.3-17.6), be referred from another healthcare unit (OR:4.5; CI95%: 2.5-8.3), have a neutrophil-to-lymphocyte ratio (NLR) > 5 (OR:4.88; CI95%: 2-11.8) and the use of antibiotics into the previous 3 months (OR:4.2; CI95%: 2.3-7.6) (Table 1). A map of the country was created showing the distribution of the prevalence of DRO infections in patients with infected burn referred from another healthcare unit (Figure 2).

**Conclusion:**

In patients with infected burn the Gram-negative organisms showed an important resistance rate to different classes of antibiotics. Use of CVC, NLR > 5, be referred from another healthcare unit and the use previous of antibiotics were risk factors for infection by DRO. The clinical outcome is affected by the bacterial resistance.

**Disclosures:**

All Authors: No reported disclosures

